# The cost-effectiveness of preventing mother-to-child transmission of HIV in low- and middle-income countries: systematic review

**DOI:** 10.1186/1478-7547-9-3

**Published:** 2011-02-09

**Authors:** Mira Johri, Denis Ako-Arrey

**Affiliations:** 1 Department of Health Administration, Faculty of Medicine, University of Montreal, Quebec, Canada; 2 Division of Global Health, Centre de Recherche du Centre Hospitalier de l'Université de Montréal, Quebec, Canada

## Abstract

**Background:**

Although highly effective prevention interventions exist, the epidemic of paediatric HIV continues to challenge control efforts in resource-limited settings. We reviewed the cost-effectiveness of interventions to prevent mother-to-child transmission (MTCT) of HIV in low- and middle-income countries (LMICs). This article presents syntheses of evidence on the costs, effects and cost-effectiveness of HIV MTCT strategies for LMICs from the published literature and evaluates their implications for policy and future research.

**Methods:**

Candidate studies were identified through a comprehensive database search including PubMed, Embase, Cochrane Library, and EconLit restricted by language (English or French), date (January 1st, 1994 to January 17^th^, 2011) and article type (original research). Articles reporting full economic evaluations of interventions to prevent or reduce HIV MTCT were eligible for inclusion. We searched article bibliographies to identify additional studies. Two authors independently assessed eligibility and extracted data from studies retained for review. Study quality was appraised using a modified BMJ checklist for economic evaluations. Data were synthesised in narrative form.

**Results:**

We identified 19 articles published in 9 journals from 1996 to 2010, 16 concerning sub-Saharan Africa. Collectively, the articles suggest that interventions to prevent paediatric infections are cost-effective in a variety of LMIC settings as measured against accepted international benchmarks. In concentrated epidemics where HIV prevalence in the general population is very low, MTCT strategies based on universal testing of pregnant women may not compare well against cost-effectiveness benchmarks, or may satisfy formal criteria for cost-effectiveness but offer a low relative value as compared to competing interventions to improve population health.

**Conclusions and Recommendations:**

Interventions to prevent HIV MTCT are compelling on economic grounds in many resource-limited settings and should remain at the forefront of global HIV prevention efforts. Future cost-effectiveness analyses can help to ensure that pMTCT interventions for LMICs reach their full potential by focussing on unanswered questions in four areas: local assessment of rapidly evolving HIV MTCT options; strategies to improve coverage and reach underserved populations; evaluation of a more comprehensive set of MTCT approaches including primary HIV prevention and reproductive counselling; integration of HIV MTCT and other sexual and reproductive health services.

## Background

Due to the availability of highly effective interventions to prevent mother-to-child transmission (MTCT), the birth of children with HIV is now rare in high-income countries. However, on a global scale, the epidemic of paediatric HIV continues to challenge disease control efforts. Worldwide, UNAIDS estimates that the number of children younger than 15 years of age living with HIV/AIDS increased from 1.6 million [95% CI: 1.4 million to 2.1 million] in 2001 to 2.5 million [95% CI: 1.7 million to 3.4 million] in 2009 [1,2]. An estimated 370 000 [95% CI: 230 000 to 510 000] children were newly infected in 2009 [2].

Virtually all HIV-infected children acquire the infection through MTCT, which can occur during pregnancy, labour and delivery, or through breastfeeding. In the absence of any intervention an estimated 15-30% of mothers with HIV infection will transmit the infection during pregnancy and delivery, and breastfeeding by an infected mother increases the risk by a further 5-20% to a total of 20-45% [3-5]. Without treatment, most HIV-infected children experience severe morbidity and early death.

The risk of MTCT has been reduced to below 2% in high-income countries by universal HIV screening of pregnant women and a suite of interventions for those identified as HIV+ that includes: (1) antiretroviral (ARV) prophylaxis in combinations of three or more drugs given to women during pregnancy and labour, and ARV prophylaxis given to the infant in the first weeks of life; (2) obstetrical interventions including elective caesarean delivery (prior to onset of labour and membrane rupture); and (3) complete avoidance of breastfeeding. Although evidence suggests that the three-pronged approach described above is clinically most efficacious, a variety of less complex strategies to prevent HIV MTCT (pMTCT) have been proposed for developing countries each with different resource requirements and levels of associated clinical benefit [6-11].

The World Health Organization (WHO) promotes a comprehensive approach to prevent MTCT based on four components: (1) primary prevention of HIV infection among women of childbearing age; (2) preventing unintended pregnancies among women living with HIV; (3) preventing HIV transmission from a woman living with HIV to her infant; and (4) providing appropriate treatment, care and support to mothers living with HIV and their children and families [12]. Recognising the multifaceted tradeoffs involved in selecting among alternative pMTCT approaches and their sensitivity to local context, current WHO technical guidelines leave considerable flexibility to decision makers at the country level [13,14]. In developing countries where virtually all HIV MTCT now occurs, constraints related to health system infrastructure, availability of trained personnel, and availability of resources are an inescapable part of decision-making. Information on the economic value of alternative pMTCT strategies can contribute to the design of evidence-based policy.

As access to services for preventing MTCT has increased worldwide, the number of children newly infected with HIV has dropped sharply. Incident cases for 2009 are down by almost one quarter as compared to five years earlier [2]- an unprecedented achievement that brings renewed hope to the global community. To build upon these successes, policies and programmes must reflect bold and intelligent choices. Our objective was to conduct a systematic review of the cost-effectiveness of interventions to prevent mother-to-child transmission (MTCT) of HIV in low- and middle-income countries (LMICs). This article presents syntheses of evidence on the costs, effects and cost-effectiveness of pMTCT strategies for LMICs from the published literature and evaluates their implications for policy and future research.

## Methods

### Data sources

To identify all published economic evaluations of interventions to prevent MTCT of HIV we searched the PubMed, Medline, Embase, Web of Science, Google Scholar, Cochrane Library, Econ Lit, National Health Service Economic Evaluation Database (NHS EES) and Latin American and Caribbean Health Sciences Literature (LILACS) databases from January 1^st^, 1994 (date of the earliest pharmaceutical HIV MTCT interventions^1,2^) to January 17^th^, 2011. An information retrieval specialist helped to develop the PubMed search string: "Cost-Benefit Analysis"[Mesh] OR "Costs and Cost Analysis"[Mesh] OR "Program Evaluation"[Mesh] OR "Cost Effectiveness"[Title] OR "Cost utility"[Title] OR "Health Care Economics and Organizations"[Mesh]) AND "HIV Seropositivity"[Mesh] OR "HIV"[title] OR "HIV"[Mesh] OR "Acquired Immunodeficiency Syndrome"[Mesh] AND "Disease Transmission, Vertical"[Mesh] OR "pmtct"[Mesh] OR "PMTCT"[Title]. Our search was restricted to articles in English and French. We supplemented the database search by checking article bibliographies for relevant studies and contacting experts to enquire about ongoing research. All candidate studies were exported to Endnote bibliographic software [15].

### Study selection

Inclusion and exclusion criteria were designed to retain all and only those studies pertaining to components 1-3 of the WHO MTCT strategy, which focus on prevention [12]. Two researchers independently reviewed the titles and abstracts of articles retrieved using the following criteria:

(i) *Studies *- All original research articles published in peer-reviewed scientific journals offering full economic evaluations of strategies to prevent MTCT of HIV in pregnant women in LMICs (as defined by the World Bank) [16] were candidates for inclusion. Cost-effectiveness, cost-benefit and cost-utility designs as defined by Drummond and colleagues [17] were all acceptable.

(ii) *Participants - *Women at risk of transmitting HIV infection to their children. This could include pregnant women or those at risk of pregnancy, regardless of HIV status.

(iii) *Interventions - *All interventions to prevent or reduce HIV MTCT, including (but not limited to) strategies for antiretroviral therapy and replacement feeding.

We excluded articles with the following characteristics:

(i) Studies focusing on high-income countries as defined by the World Bank [16]

(ii) Studies that are not original, peer-reviewed research articles (reviews, monographs and conference abstracts)

(iii) Studies of MTCT that provide only cost analyses (incomplete economic evaluations)

(iv) Studies focusing on general HIV/AIDS prevention without reference to MTCT

(v) Studies assessing the cost-effectiveness of therapies for children already infected with HIV

**Figure 1:**
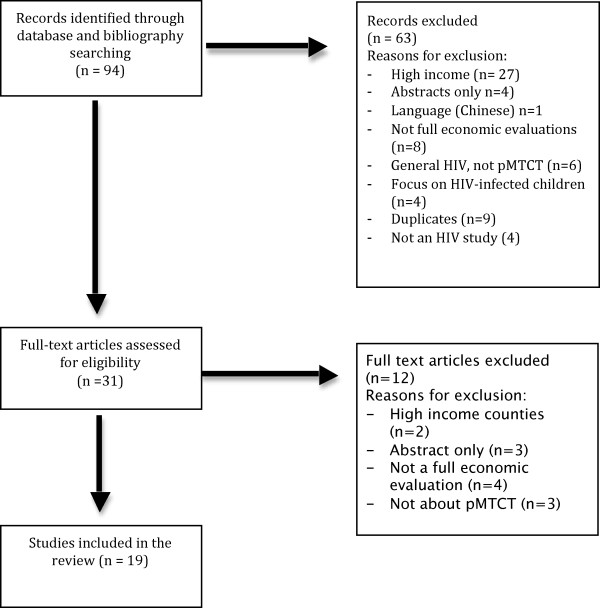
**Flow diagram of study selection**.

Review was not blinded. On the basis of initial title and abstract screening, candidate articles were retained for full text review. Articles that met the inclusion criteria were retained for data extraction. Authors jointly determined study inclusion on the basis of their individual assessments and discussion. At each stage, differences of opinion were resolved through building consensus and, in rare instances, appeal to a third reviewer [Figure 1].

### Data extraction and synthesis

Each author extracted relevant information independently using a standardised data extraction form, pre-tested on a subset of the sample. Data extraction was not blinded. Discrepancies were harmonised through building consensus. We contacted study authors with unresolved queries. Fields extracted are summarised in Tables 1, 2, 3 and 4. Due to the diversity of methodological approaches, interventions, study populations and programme comparators, we took a narrative approach to data synthesis, as is standard for systematic reviews of cost-effectiveness studies. Principal summary measures for the study are summarised in Table 4 and include cost per infant HIV infection averted, cost per life year gained, and cost per QALY or DALY.

**Table 1:** Overview of economic evaluations of interventions to reduce mother to child transmission (MTCT) of HIV

Study	**Location (Income)**^**1**^	**Adult HIV Prevalence**^**2**^	**Study Population**^**3**^	**Interventions**^**4**^	**Study design**^**5**^
[32]	SSA^6^	1% - 26%	100 000 pregnant women	(0) No intervention (1) CDC Thai	CEA
[33]	SSA	1% - 26%	100 pregnant women	(0) No intervention (1) PETRA-A (2) PETRA-B (3) PETRA-C	CEA & CUA
[29]	South Africa (UM)	18.10%	8421 pregnant women representing a **high prevalence **health district (26% HIV+)	(0) No intervention (1) ACTG 076 with breastfeeding, current infrastructure (2) ACTG 076 without breastfeeding, enhanced infrastructure (3) PETRA-A, enhanced infrastructure	CEA
[34]	SSA	1% - 26%	20 000 pregnant women	(0) No intervention (1) HIVNET 012 (targeted) (2) HIVNET 012 (universal) (3) PETRA-A (4) PETRA-B (5) CDC Thai (targeted)	CEA & CUA
[30]	South Africa (UM)	18.10%	20 000 pregnant women	(0) No intervention (1) Formula feeding (FF) recommended from birth (2) FF recommended from 4 months (3) FF recommended from 7 months (4) FF supplied from birth (5) ACTG 076 (6) PETRA-B (7) CDC Thai 8) CDC Thai + FF recommended (9) CDC Thai + FF supplied	CEA
[35]	SSA	1% - 26%	10 000 pregnant women	(0) No intervention (1) Antenatal HIVNET 012 (targeted) (2) Antenatal HIVNET 012 (universal) (3) Labour and delivery universal maternal NVP (4) Labour and delivery universal infant therapy	CEA
[36]	South Africa (UM)	18.10%	1 340 797 pregnant women (annual national average)	(0) No intervention (1) CDC Thai (targeted) + FF supplied, enhanced infrastructure	CEA
[37]	South Africa (UM)	18.10%	920 000 HIV+ pregnancies nationally over 5 years	(0) No intervention (1) 25% HIV+ pregnant women and infants receive ART^7^ (2) Strategy (1) at 75% (3) 100% pregnant women (HIV+ and HIV-) receive ART (4) 3-drug ART of 25% of non-pregnant HIV+ adults	CEA
[23]	Mexico (UM)	0.30%	958 294 pregnant women (national birth cohort)	(0) 4% VCT^8 ^to pregnant women + ACTG 076 or HIVNET 012 (1) Strategy (1) at 85% VCT (2) 30% VCT to pregnant women at highest risk + ACTG 076 or HIVNET 012 (3) VCT to HIV+ pregnant women + ACTG 076 or HIVNET 012 (4) Strategy (4) plus VCT to 15% of late presenters	CEA
[25]	SSA	1% - 26%	Simulation of national MTCT programs using data from 8 SSA countries	(0) No intervention (1) HIVNET 012	CEA & CUA
[31]	Zambia (L)	15.20%	40 000 pregnant women	Usual care (UC) = VCT + HIVNET 012 (0) UC + BF for 6 months (1) UC + BF for 12 months (2) UC + FF for 12 months (3) UC + BF for 6 months + daily infant NVP (4) VCT + Maternal 3-drug ART in pregnancy + 3-drug ART for 6 months BF (5) Same as (4), but only for women with CD4 < = 200	CUA
[27]	Thailand (LM)	1.40%	100 000 pregnant women	(0) 1 VCT + Maternal and infant ZDV as ACTG 076 (1) 1 VCT + maternal and infant NVP as HIVNET 012 (2) (1) for antenatal care + (2) for late arrivals (3) 1 VCT + combined ACTG 076 + HIVNET 012 (4) (0) with 2 VCT (5) (1) with 2 VCT (6) (2) with 2 VCT (7) (3) with 2 VCT	CEA
[22]	India (LM)	0.50%	100 000 sexually active women aged 15-49	(0) No intervention (1) Universal screening in all states + HIVNET 012 (2) Universal screening in 6 highest prevalence states + HIVNET 012	CEA & CUA
[24]	SSA	1% - 26%	100 000 sexually active women aged 15-49	(0) VCT + HIVNET 012 (5% coverage) (1) VCT + HIVNET 012 (15% coverage) (2) Family planning (contraceptive use)	CEA
[28]	South Africa (UM)	18.10%	100 000 pregnant women	For strategies 1 - 6, the analysis compared 1 VCT (base case) versus 2 VCT (1) ACTG 076 (from 28 weeks) + HIVNET 012 + ART to HIV+ve children (2) As (1) but without ART to HIV+ve children (3) ACTG 076 (from 34 weeks) + HIVNET 012 + ART to HIV+ve children (4) As (3) but without ART to HIV+ve children (5) HIVNET 012 + ART to HIV+ve children (6) Same as (5) but without ART to HIV+ve children	CUA
[26]	Kenya (L)	8.3%	10 000 pregnant women	(0) Individual VCT (1) Couple VCT	CEA
[38]	Global, results presented for 14 countries with largest numbers of HIV+ pregnant women		1 342 199 HIV+ pregnant women	(0) Antiretroviral therapy (WHO Option A antenatal & intrapartum components) (1) Strategy 0 for all HIV+ women + Family planning	CEA
[40]	Tanzania (L)	6.2%	12 747 pregnancies in catchment area in 2007 (2% HIV prevalence)	(0) No intervention (1) HIVNET 012 (2) HAART (WHO Option B)	CEA & CUA
[39]	Malawi (L)	11%	6500 pregnant women	(0) No Intervention (1) HAART (WHO Option B)	CEA & CUA

^1 ^According to the 2008 World Bank classification. LMIC = Low and Middle income countries. UM = Upper Middle Income $3,946 - $12,195; LM = Lower Middle Income ($996 - $3,945); L = Low Income ($995 or less) [16].

^2 ^Source: UNAIDS country epidemiological factsheets HIV prevalence ages 15-49 years 2009.

^3 ^Hypothetical cohorts, except for two studies [39] and [40] based on specific patient cohorts.

^4 ^Clinical trials and guidelines are described in Additional file 1. Where possible, we have numbered the base case (comparator) for the analysis as (0).

^5 ^CEA = Cost Effectiveness Analysis. CUA = Cost Utility Analysis. CBA = Cost Benefit Analysis.

^6 ^SSA = Sub-Saharan Africa

^7 ^ART = antiretroviral therapy

^8 ^VCT = voluntary counselling and testing. According to more recent terminology, all counselling and testing strategies discussed in these papers would now be referred to as "PIHT" or provider-initiated HIV testing.

**Table 2:** Economic evaluations of interventions to reduce mother to child transmission (MTCT) of HIV: study perspective and costs

Study	**Perspective**^**1**^	Cost Year & Currency	**Discount Rate**^**2**^	Cost Breakdown			
				Direct costs to the public payer			Indirect costs
					
				**Intervention costs**^**3**^	**Costs generated or offset**^**4**^	**Health system strengthening**^**5**^	
[32]	SOC	1994 US$	5%	Standard^6^	LMC^7 ^(HIV+ children)		Productivity loss due to premature mortality (HIV+^ve ^children)
	PPHC	1994 US$	5%	Standard	LMC (HIV+ children)		
[33]	PPHC	US$	5%	Standard	LMC (HIV+ children)		
[29]	PPHC	1997 US$	3%; 6%	Standard + Training		Increased health human resources	
[34]	PPHC	US$	3%	Standard	Net LMC (HIV+ children)		
[30]	PPHC	1998 US$	5%	Standard + Formula feed	Net LMC (HIV+ children)		
[35]	PPHC	1999 US	3%	Standard	LMC (HIV+ children)		
[37]	PPHC	1997 Rand	Not stated	Standard + Training			
[36]	PPHC	2000 US$	Not stated	Drugs			
[23]	PPHC	2001 US$	5%	Standard+ Formula feed Elective caesarean	LMC (HIV+ children)& HIV+ adults^8^)		
[25]	PPHC	2000 US$	3%	Standard	LMC (HIV+ children)	Human resource capacity and infrastructure	
[31]	PPHC	2003 US$	5%	Standard + Formula Feed	LMC (HIV+ children)		
[27]	PPHC	2003 US$	5%	Standard + Formula Feed	LMC (HIV+ children) Treatment costs for NVP resistance (mothers)		
[22]	PPHC	2006 Indian Rupees	5%	Standard	LMC (HIV+ children)		
[24]	PPHC	2000 US$	n/a^9^	Standard + Family planning		Program administration costs	
[28]	PPHC	2003 US$	3%	Standard + Formula Feed	LMC (HIV+ children)		
[26]	Not stated	US$	Not stated	Standard			
[38]	PPHC	US$	n/a	Standard			
[40]	Not stated	2007 US$	n/a	Standard + programme overhead			
[39]	PRO	2007 US$	3%	Standard			
	PPHC	2007 US$	3%	Standard	LMC (HIV+ children)		

^1^SOC = Societal (considers direct and indirect costs); PPHC = Public payer of healthcare costs (considers direct costs only); PRO = Provider (considers direct medical costs covered by the facility)

^2^Rates listed apply to both costs and effects.

^3^All studies included salary costs. Some were included as components of VCT while others constitute a separate category.

^4^Costs of care for HIV+ individuals averted due to the intervention or additional care required as a result of the intervention (i.e. due to adverse effects)

^5^Items considered by authors include start up costs such as training of personnel and investment in health system infrastructure, and ongoing costs such as the costs of central programme administration.

^6 ^"Standard" costs include staff time, drugs and HIV testing.

^7^LMC = lifetime medical costs

^8^Included to quantify cost savings associated with the impact of VCT on sexual behavior change and horizontal transmission

^9^n/a = non applicable

**Table 3:** Economic evaluations of interventions to reduce mother to child transmission (MTCT) of HIV: estimates of effectiveness^1^

Study	Infant HIV cases averted	**Reduction in forward transmission**^**2**^	Life years	**QALYs**^**3 **^**or DALYs**^**4**^
[32]	(0) 3764 (1) 4250 per 100,000 births^5^	n/a^6^	n/a	n/a
[33]	(1) 0.70 (2) 0.62 (3) 0.31 per 100 women	A 30% benefit was incorporated in the base case and varied from 10-50% in sensitivity analyses	n/a	(1) 13.2 (2) 11.6 (3) 5.8 DALYs per 100 women
[29]	(1) 99 (2) 272 (3) 307	n/a	n/a	n/a
[34]	(1) 476 (2) 603 (3) 315 (4) 229 (5) 309 per 20 000 women	A 30% benefit was considered in sensitivity analyses.	n/a	(1) 12572 (2) 15862 (3) 8326 (4) 6041 (5) 8163 DALYs per 20 000 women
[30]	(Total deaths averted) (1) 26 (2) 25 (3) 5 (4) 37 (5) 200 (6) 124 (7) 160 (8) 188 (9) 200	n/a	(1) 461 (2) 449 (3) 98 (4) 661 (5) 3 655 (6) 2 260 (7) 2 926 (8) 3 434 (9) 3 654	n/a
[35]	(1) 137 (2) 160 (3) 89 (4) 142	n/a	n/a	n/a
[36]	23 181	n/a		n/a
[37]	n/a	n/a	n/a	n/a
[23]	(0) 4 & 3 (1) 91 & 64 (2) 46 & 32 (3) 91 & 64 (4) 102 & 72 All reported for ACTG 076 & HIVNET 012	30% external benefit considered in sensitivity analyses	n/a	n/a
[25]	(1) Botswana: 243 Ivory Coast: 435 Kenya: 904 Rwanda: 1 380 Tanzania: 2 774 Uganda: 1 375 Zambia: 629 Zimbabwe: 1 013	n/a	n/a	(1) ^7^ BWA: 7571 CIV: 12 984 KEN: 27 784 RWA: 39 095 TZA: 82 806 UGA: 39 846 ZMB: 18 873 ZWE: 31 462 DALYs
[31]	Not given	n/a		(0) 446 208 (1) 445 922 (2) 447 391 (3) 451 250 (4) 446 869 (5) 446 187 QALYs
[27]	(0) 233 (1) 258 (2) 273 (3) 337 (4) 245 (5) 271 (6) 300 (7) 353	n/a	n/a	n/a
[22]	(1) 9880 (2) 4403	n/a	(1) 131 700 (2) 58 700 Potential years of life lost	n/a
[24]	(1) 33.1 (2) 32.5	n/a	n/a	n/a
[28]	(1) 3436 (2) 3436 (3) 3406 (4) 3406 (5) 5031 (6) 5031 For 2 VCT strategy	n/a	n/a	(1) 776.48 (2) 1158.74 (3) 1299.76 (4) 1939.63 (5) 1147.84 (6) 1712.92 QALYs for 2 VCT strategy
[26]	(1) 91 (2) 88	VCT may prevent HIV acquisition in discordant couples where the male is HIV+ve	n/a	n/a
[38]	(0) 241 596 (1) 71 945 (additional)	n/a	n/a	n/a
[40]	(1) 0.51 (2) 2.67 per 1000	n/a	n/a	(1) 12.9 (2) 67 per 1000
[39]	(1) 370	15% benefit incorporated in base case (0%-30% in sensitivity analyses)	n/a	(1) 10 449

^1^Numbers in round brackets correspond to the intervention strategies presented in Table 1.

^2^This is reduction in adult-to-adult transmission due to VCT (voluntary counseling and testing). All studies consider provider-initiated HIV testing (PIHT)).

^3^QALY = Quality-adjusted life years

^4^DALY = Disability-adjusted life years

^5^SOC = Societal (considers direct and indirect costs); PPHC = Public payer of healthcare costs (considers direct costs only); PRO = Provider (considers direct medical costs covered by the facility)

^6^n/a = not applicable

^7 ^These are three-letter country codes published by the International Organization for Standardization (ISO)

**Table 4:** Cost-effectiveness of interventions to reduce mother to child transmission (MTCT) of HIV (2008 I$)^1, 2, ^^3^

Study	Cost per infant HIV infection averted	Cost per life year	**Cost per QALY**^**4 **^**or DALY**^**5**^	**Intervention C/E? (benchmark)**^**6**^
[32]	(1) 3 748 (PPHC) (1) 1 454 (SOC)	n/a	n/a	No^**7**^
[33]	(1) 6 515 (2) 3 401 (3) 1 433	n/a	(1) 348 (2) 181 (3) 76 Cost per DALY	Yes
[29]	(1) 7 368 (2) 7 095 (3) 3 162	(1) 260; 452 (2) 251; 435 (3) 112; 194 All reported as 3%; 6% discount rate.	n/a	Yes
[34]	(1) 373 (2) 173 (3) 3 479 (4) 1 582 (5) 1 387	n/a	(1) 14 (2) 7 (3) 132 (4) 60 (5) 52 Cost per DALY	Yes (WDR^8^)
[30]	(1) 4 503 (2) 5 879 (3) 25 083 (4) 7 464 (5) 3 053 (6) 315 (7) CS^9^ (8) CS (9) 837	(1) 250 (2) 323 (3) 1 390 (4) 414 (5) 167 (6) 18 (7) CS (8) CS (9) 46	n/a	Yes (WDR)
[35]	(1) 1 044 (2) 1 021 (3) 1 196 (4) 1 021	From $5-$141	n/a	Yes
[36]	1 787	n/a	17 per DALY	Yes
[37]	n/a	(1) 23 (2) 23 (3) 163 (4) 18 363	n/a	Yes
[23]	(0) 99 430 (1) 99 318 (2) 61 286 (3) 64 732 (4) 65 733	n/a	n/a	No^**10**^
[25]	BWA: 2 022 CIV: 10 354 KEN: 4 800 RWA: 2 089 TZA: 2 554 UGA: 5 432 ZMB: 2 870 ZWE: 3 996	n/a	BWA: 65 CIV: 347 KEN: 157 RWA: 74 TZA: 86 UGA: 188 ZMB: 96 ZWE: 129 per DALY	Yes
[31]	n/a	n/a	(0) 1.96 (1) 1.98 (2) 3.25 (3) 2.98 (4) 2.46 (5) 3.60 per QALY	Yes (WDR)
[27]	(0) 716 (1) 851 (2) 570 (3) 556 (4) 1 740 (5) 1 776 (6) 1 381 (7) 1 266	n/a	n/a	Yes (Thai^12^)
[22]	(1) 1 824.61 (2) 709.30	(1) 136.91 (2) 64.18	n/a	Yes (WDR), but relative cost-effectiveness is questionable^**10**^
[24]	(1) 857 (2) 663	n/a	n/a	No^**13**^
[28]	n/a	n/a	(1) CS (2) 65 (3) CS (4) 0.5 (5) CS (6) 12.94 Incremental costs per QALY	Yes (WDR)
[26]	n/a	n/a	(0) 15.34 (1) 15.39 per DALY	Yes
[38]	(0) $543 (1) $359 (additional cost for family planning)	n/a	n/a	Yes
[40]	(1) 27 409 (2) 7 361	n/a	(1) Dominated (2) 293 per DALY	Yes/1* GDP per capita per DALY^**14**^
[39]	(1) $1010 (PRO) (1) -$267 (PPHC)	n/a	(1) $36 (PRO) (1) -$17 (PPHC) per DALY	Yes/$50 per DALY^**8 **^and 1* GDP per capita per DALY^**14**^

^1 ^To enhance comparability, all costs in this table are presented in 2008 International dollars (I$) using GDP deflators and purchasing power parities available from the International Monetary Fund [54].

^2 ^Numbers in round brackets correspond to the intervention strategies presented in Table 1. Although several studies comparing multiple strategies also provide incremental results [27-29,40], results comparing individual strategies to a do-nothing alternative are presented where possible. The exception is [28].

^3 ^SOC = Societal (considers direct and indirect costs); PPHC = Public payer of healthcare costs (considers direct costs only); PRO = Provider (considers direct medical costs covered by the facility)

^4 ^QALY = Quality-adjusted life years

^6 ^DALY = Disability-adjusted life years

^6 ^These are the study authors' conclusions about the value of one or more interventions to prevent MTCT of HIV. If a benchmark was used to justify the conclusion, it is provided in brackets.

^7 ^Study based on older (higher) drug prices and lower regimen effectiveness.

^8 ^The 1993 World Development Report: Investing in Health proposed that interventions costing less than $100 per life year saved are cost effective for middle-income countries while $50 per life-year gained is a reasonable benchmark for low-income countries [33]. This was updated to $64 per QALY in low-income settings ($50 per QALY gained, adjusted to 2003 dollars) by [26] and [29].

^9 ^CS = Cost saving

^10 ^Concentrated epidemic; very low HIV prevalence.

^11 ^Three-letter country codes published by the International Organization for Standardization (ISO). See Table 3.

^12 ^Authors used the Thai health system's thresholds for adopting health technologies as a benchmark.

^13^Authors' conclusions comparing the effectiveness of an ARV-based regimen (pMTCT component 3) to a family planning strategy (pMTCT component 1). Both strategies would likely be cost-effective using standard benchmarks.

^14 ^The WHO Commission on Macroeconomics in Health proposed that interventions costing 1*GDP per capita per DALY should be considered "very cost-effective", while those costing <3*GDP per capita should be considered "cost-effective" [44].

### Assessment of study quality

We adapted the British Medical Journal's quality assessment checklist for the conduct and reporting of economic evaluations [18], a 35-item scale that has recently been used in systematic reviews of cost-effectiveness studies [19,20]. To assess risk of bias, we included an additional item to reflect whether the article included information on sponsorship or conflict of interest. For each article, the resulting 36 items were scored as present/satisfactory, absent/unsatisfactory, or not applicable. We summed the number of absent/unsatisfactory responses to obtain a global score in which higher values represent poorer quality [21]. Quality was assessed independently by two reviewers and disagreements resolved through discussion. Quality assessment did not affect data synthesis, but did influence interpretation of results. The review protocol is available from the corresponding author.

## Results

### Study overview

We identified 19 articles published in 9 journals from 1996 to 2010, with the majority (16 of 19) focussing on sub-Saharan Africa[Table 1]. Ten studies performed only cost-effectiveness analyses (CEA), two performed only cost-utility analyses (CUA), while seven performed both CEA and CUA. No cost-benefit studies were found. All articles modelled hypothetical cohorts. Studies were conducted in a variety of epidemic contexts with HIV prevalence in pregnant women ranging from under 1% to 26%. Two studies modelled pMTCT options for 'low' or 'concentrated' epidemics, in which HIV is confined mainly to sub-populations with specific risk profiles and HIV prevalence in the general population (and, thus, pregnant women) is under 1% [22,23]. Most addressed generalised epidemics where more than 1% of the general population is HIV positive. Country income levels ranged from low to upper middle [16]. Drug regimens and related efficacy estimates were drawn from clinical trials, as was information on the natural history of MTCT [Additional file 1].

Components 1 to 3 of the WHO pMTCT strategy deal with HIV prevention [12] and related articles were included in this review. Intervention options were unequally distributed among components. Two studies considered the value of primary prevention of HIV infection among women of childbearing age (component 1) [24,25], and two articles considered prevention of unintended pregnancies among women living with HIV (component 2) [25,26]. All 19 articles considered preventing HIV transmission from a woman living with HIV to her infant (component 3). Of these, three examined different approaches to voluntary counselling and testing (VCT) [27-29]; fifteen explored alternative strategies based on antenatal, intrapartum or postpartum options using drug regimens, and three evaluated different approaches to infant feeding for prevention of postpartum transmission in the context of ART-based pMTCT [30-32]. Component 4 of the WHO strategy focuses on treatment and care rather than on prevention and related papers were excluded [22-40].

### Costs

All 19 articles considered costs incurred under the perspective of the public payer of healthcare costs[Table 2]. One study [33] also evaluated costs from a societal perspective. Costs evolved considerably during the 14-year period (1996 - 2010) over which articles were published due to a sharp drop in drug prices, shorter duration of pMTCT interventions, and increased adherence to treatment [41].

#### Intervention costs

There was considerable agreement on the components of intervention costs. All 19 studies included the costs of staff time to deliver the interventions, drugs and HIV testing, as well as additional costs specific to the interventions under study. Reported unit costs varied across studies, reflecting differences among countries in which costs were recorded, the cost year and the price of the intervention at that point in time. VCT costs ranged from $4 to $18.5 per episode; formula feeding costs were estimated at $15-$30 per month [23,30,31].

#### Costs generated or offset

Thirteen studies considered the lifetime medical costs of HIV+ children (total or net). Estimates ranged from $141 to over $11,000. One study included the lifetime cost of HIV treatment for adults [23]. One article considered costs associated with an adverse event, NVP resistance in mothers [28].

#### Health System Strengthening (HSS)

Particularly in resource-limited settings, it may be unrealistic to model the cost-effectiveness of wide scale provision of an intervention based on incremental patient costs (intervention costs at the point of delivery) at a single site with relatively developed infrastructure. We use the term "health system strengthening" to capture a variety of features not commonly considered in analyses that focus on the delivery point of interventions to patients. Three studies [24,25,30] considered the costs of HSS required to provide interventions in contexts of resource scarcity. Items considered by authors include start up costs such as training of personnel and investment in health system infrastructure, and the costs of programme administration. HSS costs resemble the category of "programme costs" as described in [42] but capture more extensive investments in physical infrastructure and health human resources.

#### Indirect costs

Productivity losses due to the early death of HIV+ children were considered by one study [33].

Choice of discount rate was quite consistent with values of 3% or 5% most commonly used.

### Effectiveness

The most common measure of effectiveness was infant HIV infections averted, reported by 17 of 19 studies[Table 3]. Four studies [23,34,35,39] considered the benefits of MTCT interventions on horizontal transmission by incorporating a reduction in adult-to-adult transmission due to VCT. No study considered the impact of pMTCT on maternal health. More general measures of effectiveness were also used. Two studies presented costs per life year gained [22,31], and seven studies presented cost per QALY [29,32] or DALY [25,34,35,39,40].

Parameter values for efficacy and effectiveness were largely drawn from clinical trials [Additional file 1]. Estimated natural history rates of MTCT of HIV in the antenatal or intrapartum period ranged from 19% to 30%. Breastfeeding transmission rates in the absence of treatment ranged from 10% to 16%. Drug efficacy reflected drug type and regimen, acceptance of testing and adherence to treatment. Acceptance of HIV testing ranged from 64% to 85% while adherence rates to anti-retroviral therapy were estimated at around 75% for ZDV and slightly over 90% for NVP.

### Cost-effectiveness

Sixteen articles concluded that an MTCT intervention was cost-effective[Table 4]. Divergent results were found by [33], which analyzed long course AZT and reflected older (higher) drug costs, and [22,23], which considered very low HIV prevalence settings. Eight articles made use of an external benchmark to justify their conclusions: six [22,29,31,32,35,39] used cutoffs for cost-effectiveness of health interventions in LMICs proposed by the World Bank [43], two [39,40] referred to the Commission on Macroeconomics and Health [44] while one [28] used standards adopted by the Thai government. The vast majority of studies included a no-intervention option in their analyses; six [23,26-29,32] did not. Many considered average rather than incremental cost-effectiveness.

Sixteen articles performed a sensitivity analysis and all found that results were sensitive to changes in at least one parameter value. The most common forms of sensitivity analysis used were one way, two way, scenario and threshold. Probabilistic sensitivity analysis was used by only five studies [25,28,29,38,40]. Cost effectiveness of pMTCT interventions was positively correlated with rates of HIV prevalence and highly sensitive to changes in this variable. Drug costs, VCT costs, natural history MTCT rate, adherence to therapy, drug efficacy, and feeding practices also had an important effect on implied optimal strategy.

### Study quality

**Figure 2:**
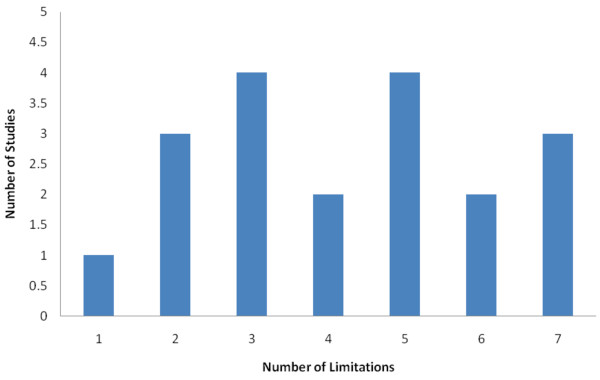
**Study Quality**. Limitations were assessed using a modified version of the BMJ quality assessment checklist for the conduct and reporting of cost-effectiveness studies [16]. A higher score reflects poorer quality.

Study quality as assessed by the BMJ checklist was poor. The number of methodological limitations in the 19 articles ranged from one to seven, and eleven studies had four limitations or more[Figure 2]. Several studies did not present the economic model adequately or did not clearly explain how outcomes were calculated. Many studies did not report potential conflicts of interest or funding source; however, those that did reported funding from not-for-profit sources.

## Discussion

We conducted a systematic review of the cost-effectiveness of interventions to prevent HIV MTCT in low- and middle-income countries. Collectively, the articles suggest that interventions to prevent paediatric infections can be cost-effective in a variety of LMIC settings as measured against accepted international benchmarks [16,44]. In concentrated epidemics where HIV prevalence in the general population is low, MTCT strategies based on universal or targeted testing of pregnant women may not compare well against cost-effectiveness benchmarks, or may satisfy formal criteria for cost-effectiveness but offer a low relative value in relation to competing interventions to improve population health.

Study conclusions can be influenced by selection of parameter values as well as methodological and modelling choices [45]. Values for epidemiological parameters related to the natural history of MTCT and intervention efficacy were frequently drawn from clinical trials (Additional file 1) and estimates of HIV prevalence from UNAIDS. Sources of costing parameters were more variable and potentially less accurate, particularly with respect to the representativeness of costs estimates drawn from specific health facilities [46]. Values for parameter inputs were generally credible and variations plausibly reflect real differences among studies. Results were sensitive to changes in key input parameters such as HIV prevalence and drug costs and advanced forms of sensitivity analysis to investigate the impact of parameter uncertainty were rarely used.

Analytical methods were fairly consistent between studies. Due to the strong scientific understanding of the natural history of HIV MTCT and the quality of the clinical evidence surrounding mechanisms to block transmission to infants, most studies focussed on interventions related to component 3 of the WHO strategy (preventing HIV transmission from a woman living with HIV to her infant) [12] and the outcome of paediatric HIV infections prevented. HIV infections prevented were translated by several studies into more general measures such as life expectancy, QALYs or DALYs. No CBA studies were found. There was considerable convergence in the choice of discount rates, although a justification was rarely provided. Moreover, as costs and health benefits were usually incurred within a very short time horizon the discount rate did not substantially influence results. The central policy choices surrounding pMTCT relate to the health care payer perspective, which was modelled by all studies. One study also considered the societal perspective; however, credible data for judging lifetime productivity gains and losses is often unavailable especially in developing countries. The most important area of analytic divergence concerned approaches to costing. The majority of studies considered only intervention costs and neglected costs related to programme start up and administration, as well as investment in health human resources and infrastructure. This is an important albeit common omission in the cost-effectiveness literature [42] that would have caused studies to overstate cost-effectiveness.

Modelling choices relate to the type of model and the structure chosen for an analysis [45]. Without exception, the 19 articles reviewed used static natural history models based on analyses of decision trees and hypothetical Markov cohorts. Many studies did not present the modelling framework in a clear and reproducible way. Modelling choices and challenges likely influenced the range of pMTCT interventions considered by analysts, as static models depicting the natural history of HIV transmission from mother to child are best suited to assess component 3 of the recommended WHO approach. Moreover, high-quality short-term effectiveness data from randomised and observational studies are available for component 3 interventions. Modelling the effects of pMTCT components 1 and 2 may demand more complex forms of model capable of capturing the dynamics of infection and transmission in the general population, as well as more comprehensive data permitting extrapolation over longer time horizons. A dynamic modelling approach is conceptually desirable and would be likely to have a significant impact on estimates of cost per HIV infection averted, the epidemiological impact of pMTCT, and choice of optimal prevention strategy [24,25]. However, it would plausibly tell in the direction of making interventions related to component 3 more cost-effective and thus would not alter the main conclusions of the review.

Limitations of this review include reliance on published articles and English or French language sources. Notwithstanding these limitations and variations in the quality of analysis and reporting in this group of papers, the general finding that short-course pMTCT interventions reflecting recent (lower) drug prices can be cost-effective in a wide variety of resource-limited contexts, with the possible exception of low HIV prevalence settings, emerges as a consistent message. The fact that interventions are highly effective and confer benefits to newborns with a long life expectancy contributes to the robustness of results across countries and makes them relatively insensitive to choice of outcome measure. The high cost of case finding relative to health benefits gained is responsible for the equivocal cost-effectiveness result in settings of low HIV prevalence. A 2003 systematic review also found that interventions for pMTCT using short course regimens such as CDC-Thai and HIVNET 012 were potentially cost-effective in sub-Saharan Africa [41].

Despite the significance of the problem and consistency of the overall message, the majority of studies model interventions of limited relevance for clinicians and policymakers due to rapid evolution in the recommended standard of care to prevent paediatric infections. Ongoing research and programme experience have helped to define new pMTCT approaches and the strategies reviewed have largely been superseded by more effective and more resource-intensive clinical options. For HIV-infected women who do not need treatment for their own health, the WHO now proposes two options designed to prevent MTCT while preserving future treatment options for the mother [13]. (Additional file 1) In addition, WHO recommends that appropriate antiretroviral therapy be given to HIV+ women who require it for their own health [13]. Two studies to date have examined the currently recommended approaches and confirm their cost-effectiveness in generalised epidemic contexts [39,40]. Models highlight the sensitivity of cost-effectiveness results to HIV prevalence [40], suggesting challenges for efficient delivery of these interventions in very low HIV prevalence settings.

## Conclusions

The development in the early to mid 1990s of new and very effective classes of antiretroviral drugs precipitated a major change in the standard of care for HIV infection, initially uniquely to the benefit of residents of wealthy countries. Early in the course of the HIV epidemic, representatives of major international agencies were persuaded by the logic of cost-effectiveness that antiretroviral treatment should be inaccessible to those infected with HIV in developing countries [47]. In reaction, many of the earlier studies reviewed were concerned to demonstrate that at least one compelling use of antiretrovirals, prevention of HIV transmission from mother to child, could be potentially cost-effective in even the poorest of settings. The policy dialogue focussed on defining effective options that would be inexpensive and feasible enough to be used in the contexts where need was greatest, and advocacy often shaped methodological choices.

The dialogue has now changed in two fundamental ways. First, there has been a general shift towards a more nuanced use of cost-effectiveness evidence. Rather than seeing cost-effectiveness as the only relevant criterion (or as an anathema), information on efficiency is increasingly viewed as one among a number of factors relevant to sound policymaking [48]. Other criteria, such as the severity of the condition and the special vulnerability of the primary beneficiary group, are also relevant [49]. Second, the World Health Organization has now defined guidelines for interventions to interrupt perinatal transmission [13]. The confluence of these factors means that the central question to be addressed by cost-effectiveness studies is no longer whether interventions to prevent vertical transmission of HIV should be offered, but rather, how best to offer the recommended standard of care.

Future cost-effectiveness analyses can help to ensure that pMTCT interventions for LMICs reach their full potential by focussing on unanswered questions in four areas.

1. Which pMTCT strategies are best in a given local context? The set of intervention options for HIV MTCT evolves rapidly and context-specific factors can affect the choice of best strategy. It will continue to be important for countries to conduct operational research to validate the cost-effectiveness of specific approaches in their setting, to ensure that pMTCT strategies that reflect current clinical guidelines [13,14] and are technically efficient are privileged. The cost-effectiveness of several important options remains to be evaluated in LMICs, among them: (i) new diagnostic technologies such as combined point of care diagnostic tests for HIV and other STDs; (ii) innovative strategies for reaching underserved populations (particularly in rural areas) such as the Mother-Baby Pack developed by UNICEF and partners to increase the uptake of more efficacious ARV prophylactic regimens for PMTCT in resource-limited settings, in line with the most recent WHO guidelines [13]; (iii) post-exposure prophylaxis for infants born to women who have not received an ante partum drug regimen; (iv) alternative laboratory strategies such as use of PCR for infant HIV testing. New diagnostic and laboratory options have the potential to figure as important cost drivers.

2. How can coverage of MTCT interventions in LMICs be improved? Despite the remarkable scientific advances of the last 15 years, pMTCT program coverage remains low in most LMICs [12]. A crucial question is hence how best to scale up programmes to reach underserved populations. This issue is particularly challenging where facility-based antenatal care attendance is low, a problem disproportionately affecting residents of rural areas. None of the studies reviewed considered the impact of programme scale on the cost-effectiveness ratio. The following questions are central: in which types of epidemic conditions is it important to reach underserved populations? Which mechanisms are most effective? What level of infrastructure is required to implement the interventions, or to scale up the interventions? [50] If strategies to reach rural, remote and underserved urban populations are not cost-effective or are less cost-effective than those for other groups, are there ethical or pragmatic reasons that they should nonetheless be implemented?

3. Can we evaluate a more comprehensive set of pMTCT options? Current models have focussed overwhelmingly on component 3 (perinatal transmission) of the recommended WHO pMTCT approach. To evaluate how funds for pMTCT should best be spent would properly require a broader framework in which the value of all 4 pillars of the strategy can be considered [12,24,25]. Since models are generally based on incremental analysis, failure to include appropriate strategies can lead to erroneous policy conclusions. Development of a comprehensive perspective poses new challenges that may require going beyond a static modelling approach. In addition to focussing on the natural history of mother-to-child transmission and interventions to block infant infection, the ideal model would also assess HIV transmission dynamics among adults to capture the value of primary prevention strategies and be capable of considering the value of early and appropriate care for adult HIV+ women and their infants.

4. How should pMTCT services best be organised and delivered to strengthen health systems and improve the health of women and children? While existing MTCT services are often offered vertically, fostering linkages to maternal, newborn and child health programmes and sexual and reproductive health programmes offers an opportunity to improve programme efficiency and equity, to further attainment of the goals set by the UN General Assembly for HIV [51], and to help fulfil the health related Millennium Development Goals (MDGs) [12]. Although examples of service integration models are beginning to emerge [52] many questions remain. What are the benefits and costs of linked services, including costs and cost-effectiveness? Which models work best in which contexts? [12,53] Answers are urgently needed. Though cost-effectiveness considerations should contribute to the design of integrated services, these questions challenge mainstream cost-effectiveness methods as they require cost-effectiveness models to assess a broad range of interventions in a comparable way and to consider the potential benefits of programme synergies [48].

Answers to these four sets of interrelated questions are likely to depend on features of the local context such as HIV epidemic type, the epidemiology of HIV and other conditions affecting the health of women and children, country resource levels, pricing of drugs and technologies, and local values. By addressing them, operational research on cost-effectiveness can play an important role in helping to realise the full potential of pMTCT interventions to prevent paediatric infections and promote the health of mother and child.

## Competing interests

The authors declare that they have no competing interests.

## Authors' contributions

MJ contributed to conception and design of the study, extraction, analysis and interpretation of the data, and drafted the manuscript. DAA contributed to conception and design of the study, extraction, analysis and interpretation of the data, and critical revision of the manuscript. Both authors read and approved the final manuscript.

## Supplementary Material

Additional file 1 **Appendix 1. Clinical trials and guidelines used to inform intervention strategies for the economic evaluations included in this review**. Overview of clinical trials and guidelines used to inform cost-effectiveness studies included in the review Click here for file
