# Supplementary material for: The cost-effectiveness of preventing mother-to-child transmission of HIV in low- and middle-income countries: systematic review
Source: Cost Eff Resour Alloc. 2011 Feb 9;9:3. doi: 10.1186/1478-7547-9-3 (PMC3045936; doi:10.1186/1478-7547-9-3)
Supplement: Additional file 1 — Appendix 1. Clinical trials and guidelines used to inform intervention strategies for the economic evaluations included in this review. Overview of clinical trials and guidelines used to inform cost-effectiveness studies included in the review [file 1478-7547-9-3-S1.DOC]

| **Study, Location, & Year** | **Drugs**^2^ | **Antenatal and Intrapartum** | **Postpartum** | **Mode of Infant feeding** | **Infant HIV infections prevented** |
| --- | --- | --- | --- | --- | --- |
| **PACTG 076; USA & France; 1994 [1]** | ZDV vs. Placebo | Long course (from 14 weeks gestation) + intravenous intrapartum | Long course (6 weeks) infant only | Replacement  feeding | 66% efficacy at 18 months |
| **CDC-Thai; Thailand; 1996 [2]** | ZDV vs. Placebo | Short course (from 36 weeks intrapartum) | None | Replacement feeding | 51% efficacy at 6 months |
| **RETRO-CI; Cote d’Ivoire 1996 [3,4]** | ZDV vs. Placebo | Short course (from 36 weeks intrapartum) | None | Breastfeeding | 44% after 3 months |
| **DITRAME; Cote d’Ivoire & Burkina Faso; 1996 [3,5]** | ZDV vs. Placebo | Short course (from 36 weeks intrapartum) | Short (one week) maternal only | Breastfeeding | 38% after 6 months |
| **PETRA; South Africa, Uganda & Tanzania; 1996 [6]** | ZDV + 3TC  vs.  Placebo | Short course (from 36 weeks and intrapartum) | Short course (one week), maternal and infant | Breastfeeding | 63% at 6 weeks and  15% at 18 months. |
| **HIVNET 012; Uganda; 1997 [7,8]** | NVP vs. ZDV | No antenatal ARV.  Intrapartum (single-dose NVP vs. ZDV) | Single dose (1 week) infant only | Breastfeeding | NVP 47% efficacy at 14-16 weeks |
| **SIMBA; Rwanda & Uganda ; 2001-2002 [9]** | 3TC or NVP during BF | Short course (from 36 weeks) ZDV + DDI | ZDV + DDI (1 week mother only)  3TC or NVP for infants during 3-6 months BF | Breastfeeding | HIV infection at 6 months, 8% (6% at birth, 2% postnatal . NVP & 3TC similar efficacy). |

Appendix 1. Clinical trials and guidelines used to inform intervention strategies for the economic evaluations reviewed^1^

**Clinical Trials**

^1^ Based on ‘’Antiretroviral drugs for treating pregnant women and prevention HIV infection in infants: guidelines on care, treatment and support for women living with HIV/AIDS and their children in resource-constrained settings’’ [10]

^2^ Zidovudine (AZT); Didanosine (DDI); Nevirapine (NVP); Lamivudine (3TC)

**Current WHO Guidelines to prevent MTCT for HIV-infected pregnant women who do not need treatment for their own health**

|  | **Drugs**^2^ | **Antenatal and Intrapartum** | **Postpartum** | **Mode of Infant feeding** |
| --- | --- | --- | --- | --- |
| **Option A**  **Maternal AZT + infant ARV prophylaxis** |  | Long course antenatal AZT (from 14 weeks gestation) + intrapartum single dose NVP | Maternal: 7 day AZT+3TC  Infant with BF: Daily NVP from birth until 4 to 6 weeks, and until 1 week after breastfeeding has ended.  Infant with replacement feeding: Daily NVP or sd-NVP + twice-daily AZT from birth to 4-6 weeks | Open |
| **Option B:**  **Maternal Triple ARV Prophylaxis (HAART)** | Recommended HAART  regimens include:  AZT + 3TC + LPV/r, AZT + 3TC + ABC, AZT + 3TC + EFV or TDF + 3TC (or FTC) +EFV | Long course HAART (from 14 weeks intrapartum) | Maternal: until delivery or, if breastfeeding, until 1 week after all exposure to breast milk has ended  Infant: Daily NVP or twice-daily AZT from birth until 4 to 6 weeks. | Open |

^1^ Based on “World Health Organization (2010), Antiretroviral drugs for treating pregnant women and preventing HIV infection in infants: recommendations for a public health approach. – 2010 version (Geneva: World Health Organization)” [11]

^2^ Zidovudine (AZT) ; Lamivudine (3TC) ; Lopinavir/ritonavir (LPV/r) ; Abacavir (ABC) ; Efavirenz (EFV) ; Tenofovir disoproxil fumarate (TDF) ; Emtricitabine (FTC) ; Nevirapine (NVP) ; Single-dose nevirapine (sd-NVP)

**Appendix 1 References**

1. Connor, E. M., R. S. Sperling, et al. (1994). "Reduction of maternal-infant transmission of human immunodeficiency virus type 1 with zidovudine treatment. Pediatric AIDS Clinical Trials Group Protocol 076 Study Group." N Engl J Med 331(18):1173-80.

2. Shaffer, N., R. Chuachoowong, et al. (1999). "Short-course zidovudine for perinatal HIV-1 transmission in Bangkok, Thailand: a randomised controlled trial. Bangkok Collaborative Perinatal HIV Transmission Study Group." Lancet 353(9155):773-80.

3. Wiktor, S. Z., E. Ekpini, et al. (1999). "Short-course oral zidovudine for prevention of mother-to-child transmission of HIV-1 in Abidjan, Cote d'Ivoire: a randomised trial." Lancet 353(9155):781-5.

4. Leroy V et al. (2002). "Twenty-four month efficacy of a maternal short-course zidovudine regimen to prevent mother-to-child transmission of HIV-1 in West Africa. AIDS, 16(4):631-641.

5. Dabis, F., P. Msellati, et al. (1999)."6-month efficacy, tolerance, and acceptability of a short regimen of oral zidovudine to reduce vertical transmission of HIV in breastfed children in Cote d'Ivoire and Burkina Faso: a double-blind placebo-controlled multicentre trial. DITRAME Study Group. DIminution de la Transmission Mere-Enfant." Lancet 353(9155):786-92.

6. The Petra study team (2002). Efficacy of three short-course regimens of zidovudine and lamivudine in preventing early and late transmission of HIV-1 from mother to child in Tanzania, South Africa, and Uganda (Petra study): a randomised, double-blind, placebo-controlled trial. Lancet, 359(9313):1178-86.

7. J.Jackson., P.Musoke, et al (2003). Intrapartum and neonatal single-dose nevirapine compared with zidovudine for prevention of mother-to-child transmission of HIV-1 in Kampala, Uganda: 18-month follow-up of the HIVNET 012 randomised trial. Lancet, 362(9387):859-868.

8. Guay, L. A., P. Musoke, et al. (1999). "Intrapartum and neonatal single-dose nevirapine compared with zidovudine for prevention of mother-to-child transmission of HIV-1 in Kampala, Uganda: HIVNET 012 randomised trial." Lancet 354(9181):795-802.

9. Vyankandondera J et al. Reducing risk of HIV-1 transmission from mother to infant through breastfeeding using antiretroviral prophylaxis in infants (Simba study). 2nd IAS Conference on HIV Pathogenesis and Treatment, Paris, abstract LB7, 2003

10. World Health Organization: Antiretroviral drugs for treating pregnant women and preventing HIV infection in infants: Guidelines on care, treatment and support for women living with HIV/AIDS and their children in resource-constrained settings. Geneva: World Health Organization; 2004.

11. World Health Organization (2010), Antiretroviral drugs for treating pregnant women and preventing HIV infection in infants: recommendations for a public health approach. – 2010 version (Geneva: World Health Organization).
